# Dynamics of DNA Replication during Premeiosis and Early Meiosis in Wheat

**DOI:** 10.1371/journal.pone.0107714

**Published:** 2014-10-02

**Authors:** María-Dolores Rey, Pilar Prieto

**Affiliations:** Plant Breeding Department, Institute for Sustainable Agriculture, Agencia Estatal Consejo Superior de Investigaciones Científicas (CSIC), Córdoba, Spain; Leibniz-Institute of Plant Genetics and Crop Plant Research (IPK), Germany

## Abstract

Meiosis is a specialised cell division that involves chromosome replication, two rounds of chromosome segregation and results in the formation of the gametes. Meiotic DNA replication generally precedes chromosome pairing, recombination and synapsis in sexually developing eukaryotes. In this work, replication has been studied during premeiosis and early meiosis in wheat using flow cytometry, which has allowed the quantification of the amount of DNA in wheat anther in each phase of the cell cycle during premeiosis and each stage of early meiosis. Flow cytometry has been revealed as a suitable and user-friendly tool to detect and quantify DNA replication during early meiosis in wheat. Chromosome replication was detected in wheat during premeiosis and early meiosis until the stage of pachytene, when chromosomes are associated in pairs to further recombine and correctly segregate in the gametes. In addition, the effect of the *Ph1* locus, which controls chromosome pairing and affects replication in wheat, was also studied by flow cytometry. Here we showed that the *Ph1* locus plays an important role on the length of meiotic DNA replication in wheat, particularly affecting the rate of replication during early meiosis in wheat.

## Introduction

Meiosis is a specialised type of cell division common to sexually developing eukaryotes that generates four haploid gametes from a single diploid cell. The evolutionary trends of cell cycle including DNA replication, growth control and cell division are mechanistically well conserved among eukaryotes [1]–[5]. During the cell cycle, proliferating cells pass through four stages: G1, the cell growths and the nucleus has a 2C DNA content (where C is the DNA content of a haploid genome with chromosome number n); S, DNA replicates (2C → 4C); G2, a second growth period during which the nucleus retains a 4C content until the last phase; and M, mitosis or meiosis in somatic or germinal cells, respectively, when genetic material is divided into two daughter nuclei (4C → 2C). During meiosis a second division occurs and four haploid cells (gametes) are finally obtained from one initial diploid cell.

Duplication of the genome during S phase of the cell cycle is a highly organised process, usually followed in germinal cells by chromosome pairing of homologous (identical) chromosomes, recombination and synapsis [6]. Pre-meiotic DNA replication has been shown to be similar to pre-mitotic S phase in many aspects [7]–[9] although several important features distinguish meiotic from mitotic replication, including the trigger that initiates the process [10]. In addition, pre-meiotic S phase is on average 2–3 times longer than pre-mitotic S-phase in all organisms studied [11]–[13], probably because necessary interactions between homologues for their successful recombination and segregation are initiated during pre-meiotic S phase [6], [14], [15]. Additional periods of DNA synthesis have also been reported during early meiosis in leptotene, zygotene and pachytene [16]–[18]. In fact, detection of replication during early meiosis was essential for understanding the mechanism of crossing-over during recombination [19], [20].

Pre-meiotic replication has been found to be connected to later events occurring in meiosis such as recombination and reductional chromosome segregation [21], [22]. Moreover, replication has also been shown to be closely connected temporally to chromosome condensation at the onset of meiosis [23]. Most of the studies about pre-meiotic replication have been conducted in yeast [24] and little is known about meiotic replication in plants. Replication has been recently studied during early meiosis in wheat-rye hybrids in the presence and in the absence of the *Ph1* locus [25]. Wheat (*Triticum aestivum* L.) is a staple food for most of the world population, and understanding its genetics and genome organisation is of great value for genetics and plant breeders. The *Ph1* locus controls homologous chromosome pairing in wheat [26]–[28], and has been defined to a cluster of kinase-like genes containing a segment of heterochromatin [29], [30]. Cyclin dependent kinases (CDKs) play an important role in the cell cycle regulation and transcription control [31]. The *Ph1*-like gene in wheat shares some homology to Cdk2 in mammals, which regulates the progression of replication through controlling chromatin decondensation during S phase [32]. In wheat, the *Ph1*-like gene regulates premeiotic replication, chromatin condensation, transcription of the earliest meiotic gene (Asy1), homologue pairing/synapsis, resolution of incorrect pairing at pachytene and recombination [33]. Recent studies have described that the *Ph1* locus may affect replication through either an increment in the activation of origins and hence the rate of replication of the dispersed chromatin or, a delay in the initiation of heterochromatin replication in the absence of the *Ph1* locus [25].

Flow cytometry has become a useful method for studying the characteristics of eukaryotic cells, with applications in crop and horticultural science [34]. Although flow cytometry has been crucial for chromosome sorting, allowing sequencing in species with large genomes such as wheat [35], other popular flow cytometric applications are the measurement of cellular DNA content for studies of ploidy, mostly in plants, and the identification of the cell distribution during the cell cycle [36]–[38]. In fact, cell cycle-phase distribution of the DNA synthesis activity can be effectively determined by flow cytometry after isolation of nuclei. The four distinct phases (G1-, S-, G2- and M) can be recognised in a proliferating cell population by flow cytometry, although G2- and M-phase, which both have an identical DNA content (4C), can not be discriminated based only on their differences in DNA content. Therefore cytogenetic approaches are required to determine whether chromosomes have entered meiosis by visualising chromosome condensation and pairing.

In this work we aimed to further our knowledge of pre-meiotic and meiotic replication in wheat, focusing in the early meiosis stages using flow cytometry. To achieve this, we established a quick and user-friendly flow cytometry-based method to investigate replication during meiosis in wheat through the quantification of the amount of DNA in each meiotic stage. Flow cytometry has been revealed as a rapid and robust method to quantify the amount of DNA during the five sub-stages (leptotene, zygotene, pachytene, diplotene and diakinesis) of early meiosis (prophase I) in wheat, and allowed a correlation between the amount of DNA and the level of replication at each stage during early meiosis in bread wheat. In addition, the effect of the *Ph1* locus on the timing and on the rate of replication during early meiosis in wheat is also discussed.

## Materials and Methods

### Plant material

Seeds of bread wheat (*Triticum aestivum* L., 2n = 2x = 42) cv. *Chinese Spring* (CS) in the presence and in the absence of the *Ph1* locus were kindly provided by Dr. Steve Reader from The John Innes Centre (Norwich, U.K.). DNA from wheat lines either in the presence or in the absence of the *Ph1* locus were extracted from young frozen leaf tissue using the CTAB method [39] with some modifications [40]. CS and CS *ph1* mutants were checked for the *ph1* deletion using the ABC_920_ SCAR marker as described previously [41].

Seeds were germinated in the dark at 25°C on moistened filter paper in petri dishes for 2 days and then transferred into pots and grown in the greenhouse at 26°C during the day and 22°C at night with a photoperiod of long days (16 h of daylight).

### Preparation of samples for flow cytometry and in situ hybridisation

Spikes were collected from plants entering meiosis, and fixed in 100% ethanol: acetic acid (3∶1, v/v) for at least one week. Florets from fixed wheat spikes were checked under a phase-contrast microscope (PrimoStar light microscope; Carl Zeiss, Göttingen, Germany) for correct assignment of the meiotic stage. Each floret has three synchronous anthers, thus one anther per floret was squashed in 45% acetic acid in water and assigned to each meiotic stage by observation under a PrimoStar light microscope (Carl Zeiss, Göttingen, Germany). The two remaining anthers were fixed in 100% ethanol: acetic acid 3∶1 (v/v) and used for flow cytometric analysis and *in situ* hybridisation. Young leaves from both wheat lines were used as somatic control in flow cytometry experiments.

### In situ hybridisation

Fixed anthers were squashed in 45% acetic acid in water for *in situ* hybridisation. The telomeric sequence was amplified by PCR using the (5′-TTTAGGG-3′) and (5′- CCCTAAA-3′) primers in the absence of template DNA [42] and a cereal centromeric sequence (CCS1) was amplified using the conditions described by [43]. The *in situ* hybridisation protocol was performed according to [44]. Digoxigenin-labelled centromeres and biotin-labelled telomeres were detected with antidigoxigenin-FITC (Roche Applied Science, Indianapolis, IN, USA) and streptavidin- Cy3 conjugates (Sigma, St. Louis, MO, USA), respectively. Chromosomes were counterstained with DAPI (4′, 6-diamidino-2-phenylindole) and mounted in Vectashield. Hybridisation signals were visualised using a Nikon eclipse 80i epifluorescence microscope. Images were captured with a Nikon CCD camera using the Nikon 3.0 software (Nikon Instruments Europe BV, Amstelveen, The Netherlands) and processed with Photoshop 4.0 software (Adobe Systems Inc., San Jose, California, USA).

### Flow cytometric analysis

Wheat anthers at each meiotic stage were ground with a pestle in 400 µl nuclear extraction buffer of the Partec CyStain UV Precise T kit (PARTEC GmbH, Münster, Germany) for 3 min at room temperature following the instructions of the supplier. The suspension was filtered through a 30 µm nylon mesh filter to discard cell debris. Finally, each sample was stained with 4′, 6-diamidino-2-phenylindole (DAPI) for 1 min to measure the amount of nuclear DNA using a CyFlow Ploidy Analyser (PARTEC GmbH, Münster, Germany) equipped with an UV LED. Three independent experiments, consisting of the measurement of the DNA content of samples in leptotene, zygotene, pachytene, diplotene, diakinesis and metaphase I either in the presence or in the absence of the *Ph1* locus, were carried out on different days. In addition, 3 replicate measurements of each sample were taken for each biological replicate. At least 5000 nuclei were counted in each sample either in the presence or in the absence of the *Ph1* locus and the coefficient of variation (CV) for each sample was always under 8.0%. The histograms were analysed using the Cylchred Software from Cardiff University developed by Terry Hoy, which is a cell cycle analysis software based on previously developed algorithms [45], and allows removing the cell debris marker from the histograms.

### Statistical analyses

Statistical analyses were performed using STATISTIX 9.0 software (Analytical Software, Tallahassee, FL, USA). The analysis of variance (ANOVA) was based on randomised blocks. Means were separated using the Least Significant Difference (LSD) test with a probability level of 0.05.

## Results

### Identification, isolation and flow cytometric analysis of meiocytes during premeiosis and early meiosis in wheat

To study the progression of replication during early meiosis in wheat, one anther per floret was carefully checked to determine the meiotic stage using a light microscopy. Since all the anthers in the same flower are synchronised, the two remaining anthers were stored in 100% ethanol: acetic acid (3∶1, v/v) at 4°C. The identification, selection and isolation of anthers was carried out until a total of 150 anthers were accumulated in each meiosis stage of prophase I (leptotene, zygotene, pachytene, diplotene and diakinesis) and metaphase I, either in the presence or in the absence of the *Ph1* locus. Each sample was then separated in three aliquots of 50 anthers each with the aim of having three independent replicates of each stage of meiosis, either in the presence or in the absence of the *Ph1* locus for three independent experiments. In addition, three different flow cytometric measurements were taken from each sample in each experiment to account for equipment deviations.

**Figure 1:**
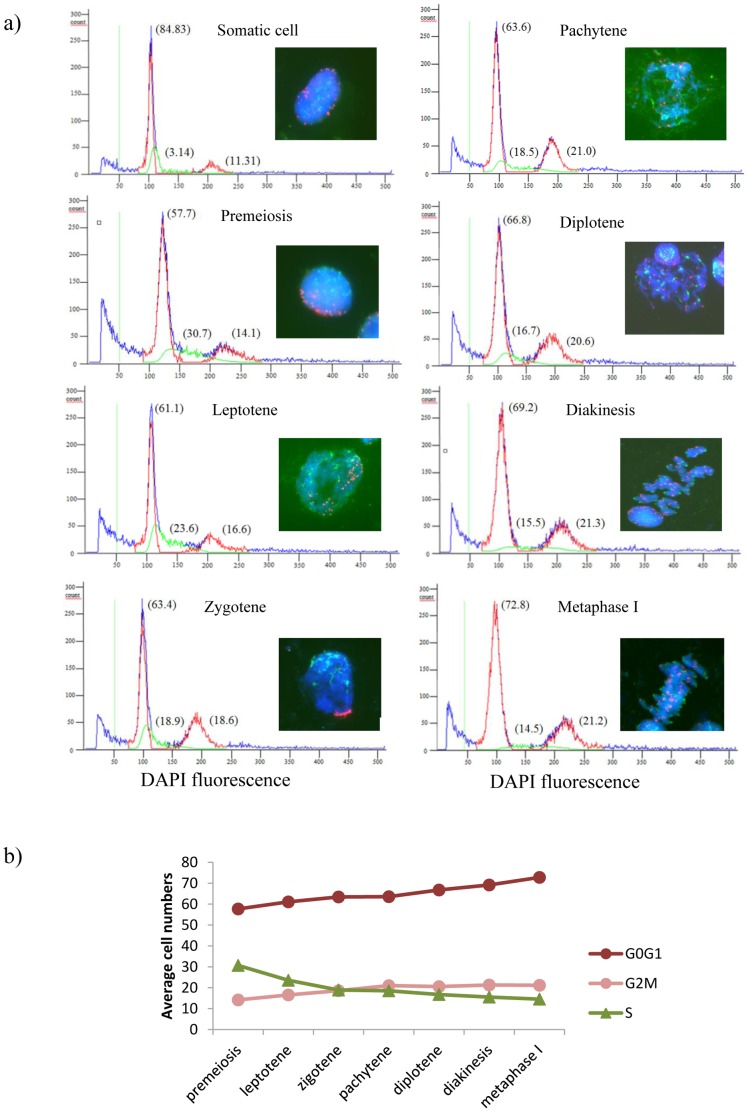
Flow cytometric analysis of DNA replication in wheat anthers during early meiosis in the presence of the *Ph1* locus. a) Flow cytometric histograms of the nuclear DNA content of isolated anther nuclei in each stage of meiosis, which was determined by the number and organisation of centromeres (green) and telomeres (red) using fluorescence *in situ* hybridisation. The percentage of cells in G0/G1, S and G2/M are in brackets. G0/G1 and G2/M phases are shown in red, S phase is shown in green and total histogram (measured as total nuclei) is shown in blue. b) Progression of the percentage of cells in G0/G1, S and G2/M phases during early meiosis.

**Figure 2:**
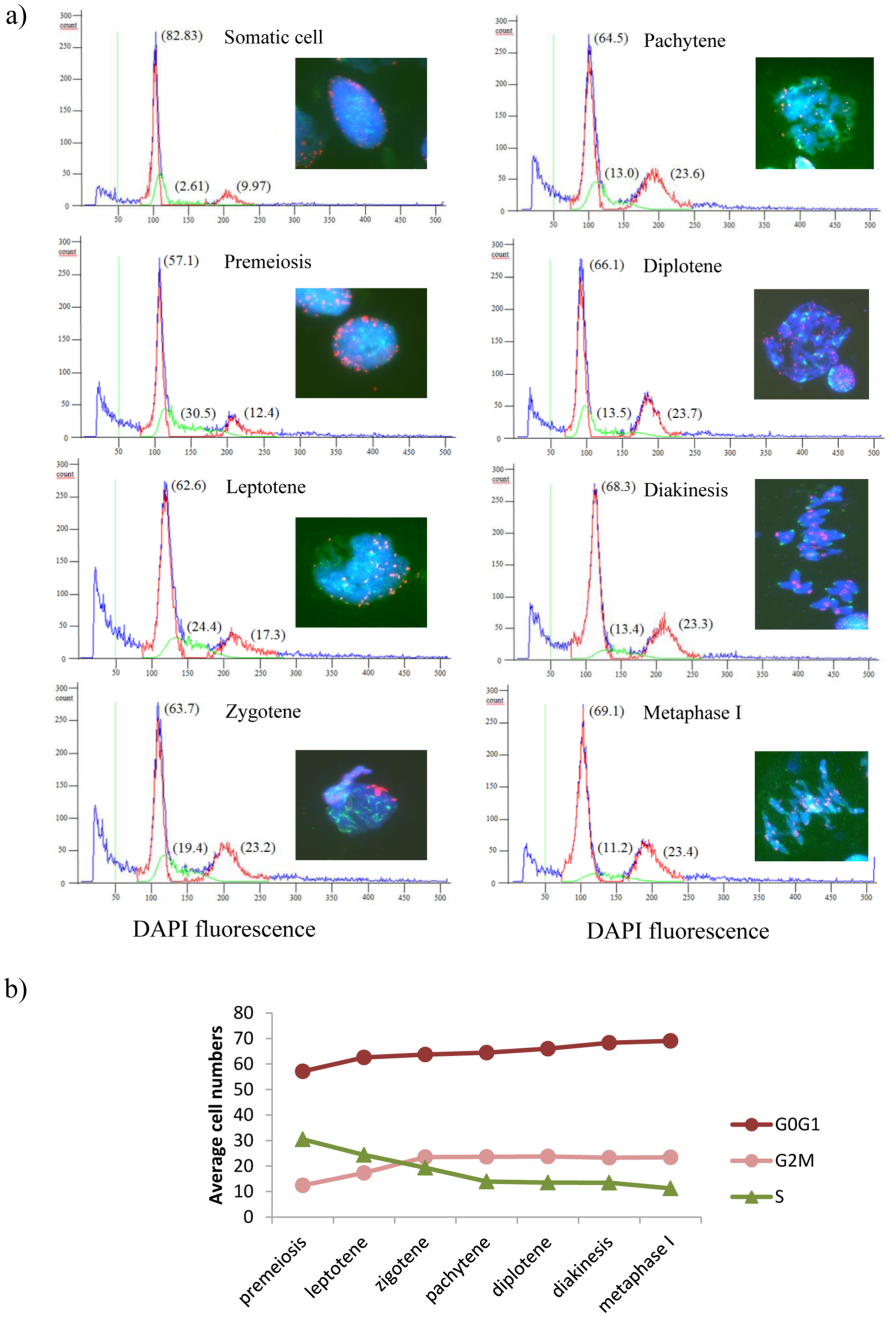
Flow cytometric analysis of DNA replication in wheat anthers during early meiosis in the absence of the *Ph1* locus. a) Flow cytometric histograms of the nuclear DNA content of isolated anther nuclei in each stage of meiosis, which was determined by the number and organisation of centromeres (green) and telomeres (red) using fluorescence *in situ* hybridisation. The percentage of cells in G0/G1, S and G2/M are in brackets. G0/G1 and G2/M phases are shown in red, S phase is shown in green and total histogram (measured as total nuclei) is shown in blue. b) Progression of the percentage of cells in G0/G1, S and G2/M phases during early meiosis.

Flow cytometric determination of the nuclear DNA content in a wheat anther sample, either in the presence or in the absence of the *Ph1* locus, was distributed in a histogram with two peaks corresponding to G0/G1 phases (un-replicated cells; 2C DNA content) and G2/M phases (replicated cells; 4C DNA content), respectively (Figures 1 and 2). As expected, most of the cells in each meiosis stage were identified in the 2C peak (G0/G1) for all the samples analysed (Figures 1 and 2). The small peak (4C) corresponded to those cells that had already finished replication and cells going under active replication were detected between the 2C and 4C peaks (Figures 1 and 2).

The amount of DNA during the cell cycle in somatic tissues was always measured by flow cytometry at the beginning and at the end of each experiment for both wheat lines (*Ph1*+ and *Ph1*-), to have a basal reference of unreplicated cells, replicated cells and somatic replication of each wheat line and, in addition, to monitor instrument or staining variations (Figures 1 and 2). As expected, most of the cells were in G0/G1 stage and no significant differences were found in wheat somatic tissue in the presence and in the absence of the *Ph1* locus in any case (Table 1). The replication value obtained for the somatic tissue (3.1±0.2 and 2.6±0.6 in *Ph1*+ and *Ph1*- wheat lines, respectively) was always at least five times lower than the minimum replication value obtained in early meiosis (15.5±1.2 and 13.5±0.1 in diakinesis in *Ph1*+ and *Ph1*- wheat lines, respectively) (Tables 2 and 3).

**Table 1:** Flow cytometric determination of the percentage distributions of wheat somatic nuclei in each phase of the cell cycle in the presence and in the absence of the *Ph1* locus (*Ph1*+ and *Ph1*-, respectively).

	G0/G1 phase		S phase		G2/M phase	
	Mean ±SE	CV	Mean ±SE	CV	Mean ±SE	CV
***Ph1+***	84.8±4.1a	4.8	3.1±0.2a	3.5	11.3±0.4a	3.8
***Ph1-***	82.8±3.1a	3.7	2.6±0.6a	3.9	10.0±0.6a	5.8

G0/G1 and G2/M values correspond to un-replicated and post-replicated cells, respectively. S phase values correspond to cells under active replication. Values are given as a mean of 9 measures, standard error of the mean (SE) and coefficient of variation (CV). The same letter indicates that there is no difference among treatments (*Ph1*+ and *Ph1*-) within the same cell cycle phase (G0/G1, G2/M and S) in somatic cells at P<0.05.

**Table 2:** Flow cytometric determination of the percentage distributions of nuclei from wheat anthers in each phase of the cell cycle during meiosis in the presence of the *Ph1* locus.

	G0/G1 phase		S phase		G2/M phase	
	Mean ±SE	CV	Mean ±SE	CV	Mean ±SE	CV
**Somatic cells**	84.8±4.1a	4.8	3.1±0.2a	3.5	11.3±0.4a	3.8
**Premeiosis**	57.7±2.3b	3.9	30.7±1.2b	3.8	14.1±4.0b	0.3
**Leptotene**	61.1±1.7bc	2.7	23.6±1.4c	6.0	16.6±0.8c	5.0
**Zygotene**	63.4±1.4cd	2.2	18.9±1.0d	5.3	18.6±0.4d	2.3
**Pachytene**	63.6±0.2cd	0.3	18.5±0.8de	4.5	21.0±1.0e	5.0
**Diplotene**	66.8±0.2de	0.3	16.7±0.4de	2.6	20.6±1.3e	6.1
**Diakinesis**	69.2±0.2ef	0.4	15.5±1.2de	7.7	21.3±0.6e	3.1
**Metaphase I**	72.8±0.3f	0.4	14.5±0.6e	4.4	21.8±1.5e	6.9

G0/G1 and G2/M values correspond to un-replicated and post-replicated cells, respectively. S phase values correspond to cells under active replication. Values are given as a mean of 9 measurements, standard error of the mean (SE) and coefficient of variation (CV). The same letter indicates no differences among treatments (stages of meiosis) within the same cell cycle phase (G0/G1, G2/M and S) at P<0.05.

**Table 3:** Flow cytometric determination of the percentage distributions of nuclei from wheat anthers in each phase of the cell cycle during meiosis in the absence of the *Ph1* locus.

	G0/G1 phase		S phase		G2/M phase	
	Mean ±SE	CV	Mean ±SE	CV	Mean ±SE	CV
**Somatic cells**	82.8±3.1a	3.7	2.6±0.6a	3.9	10.0±0.6a	5.8
**Premeiosis**	57.1±0.2b	0.4	30.5±0.9b	2.8	12.4±0.6b	5.0
**Leptotene**	62.6±0.7c	1.1	24.4±1.1c	7.9	17.3±0.2c	4.1
**Zygotene**	63.7±0.1cd	0.4	19.4±0.5d	2.5	23.2±0.5d	2.2
**Pachytene**	64.5±0.4d	1.5	13.9±0.9e	6.5	23.6±1.4d	5.9
**Diplotene**	66.6±0.3e	2.5	13.5±0.7f	4.3	23.7±0.3d	1.0
**Diakinesis**	68.3±0.6f	1.9	13.5±0.1f	0.3	23.3±1.3d	5.5
**Metaphase I**	69.1±0.6g	0.9	11.2±0.7g	6.1	23.4±1.5d	2.5

G0/G1 and G2/M values correspond to un-replicated and post-replicated cells, respectively. S phase values correspond to cells under active replication. Values are given as a mean of 9 measurements, standard error of the mean (SE) and coefficient of variation (CV). The same letter indicates no differences among treatments (stages of meiosis) within the same cell cycle phase (G0/G1, G2/M and S) at P<0.05.

### Dynamics of replication during early meiosis in hexaploid wheat

Flow cytometric analysis was carried out in wheat anthers to establish the temporal sequence of replication during early meiosis in wheat. To correctly stage the meiocytes during early meiosis, *in situ* hybridisation was carried out to allow the visualisation of chromosome dynamics by labelling centromeres and telomeres (Figure 1a). At the onset of meiosis most of the cells were located in G0/G1 phase (2C DNA content), which mostly corresponded to the somatic cells surrounding the meiocytes in the anther (Figure 1). The number of cells in G0/G1 phase slightly increased as meiosis progressed (Figure 1, Table 2), as a consequence of the anther cells multiplication to enlarge the anther size.

Interestingly enough, the number of cells under active replication in wheat anthers in premeiosis was much higher and significantly different than in the somatic control (30.7+1.2 and 3.1+0.2, respectively) (Table 2). In fact, the level of replication in wheat anthers in premeiosis was almost ten times higher than in the somatic tissue which reveals that replication is occurring during premeiosis in wheat. In addition, replicating cells (S value) were also detected in leptotene, zygotene and pachytene (Figure 1, Table 2). The replication values decreased from premeiosis (30.7+1.2) to pachytene (18.5+0.8). Then, replication remained constant from pachytene to diakinesis, but higher (15.5+1.2) than the replication value obtained for the somatic control (3.1+0.2). These results suggested that residual synthesis of DNA occurred in wheat after pachytene, when chromosomes are already associated in pairs.

According to these results, the number of cells already replicated in G2/M (4C DNA content) was higher and statistically different in premeiosis than in the somatic tissue (14.1+4.0 and 11.3+0.4 respectively), which correlates with replication in premeiosis in wheat anther. At the onset of meiosis the number of replicated cells increased from premeiosis (14.1+4.0) up to pachytene (21.0+1.0), being 1.9 times higher in pachytene than in the somatic control and confirming that replication actively occurs during early meiosis in wheat (Figure 1, Table 2). The number of replicated cells from pachytene to metaphase I remained constant and almost double compared with the somatic control (21.8+1.5 and 11.3+0.4, respectively) (Table 2). These results confirm that replication occurs during early meiosis in wheat, as an increment in the number of replicated cells was clearly detected from premeiosis to pachytene. Hence, flow cytometry is an efficient tool to successfully detect and quantify replication during early meiosis in wheat, and shows that replication occurs actively from premeiosis until pachytene, when chromosomes are paired and telomeres are clustered at the bouquet.

### Dynamics of replication during early meiosis in hexaploid wheat in the absence of the Ph1 locus

Replication was also studied by flow cytometry in early meiosis in wheat in the absence of the *Ph1* locus, which affects replication and controls chromosome pairing during meiosis. Chromosome dynamics was tracked using *in situ* hybridisation during early meiosis by labelling centromeres and telomeres to correctly stage meiosis (Figure 2a). As expected, most of the cells detected by flow cytometry corresponded to the G0/G1 cell cycle phase (2C peak) in all the stages analysed (Figure 2, Table 3). The number of cells in G0/G1 phase increased as long as meiosis progressed, consequence of the increment in the number of the somatic cells surrounding the meiocytes as the anther growths (Figure 2, Table 3).

Cells going under active replication (S phase) were also clearly detected in early meiosis in wheat in the absence of the *Ph1* locus using flow cytometry (Figure 2). In fact, the number of cells detected in replication was 11.7 times higher in premeiosis than in the somatic cell control in the absence of the *Ph1* locus (30.5+0.9 and 2.6+0.6, respectively) (Table 3). Replication was also detected in leptotene and zygotene 9 and 7.5 times higher respectively than in the somatic control. Thus, replication decreased sharply from premeiosis until reaching zygotene (Table 3). The level of replication remained constant after zygotene but slightly higher than the somatic cell control (Table 3), suggesting that residual synthesis of DNA also occurred after zygotene in wheat anther in the absence of the *Ph1* locus. Therefore, active replication was detected by flow cytometry in wheat in the absence of the *Ph1* locus, with particularly high levels of replication in premeiosis and in early meiosis (leptotene and pachytene).

The number of cells in the G2/M phases of the cell cycle corresponding to replicated cells was significantly higher in premeiosis than in the somatic control (12.4+0.6 and 10.0+0.6, respectively). Moreover, the number of replicated cells did also increase during leptotene up to zygotene, where the level of replicated cells detected was almost double the number of replicated cells in premeiosis (23.2+0.5 and 12.4+0.6, respectively; Figure 2a, Table 2). Finally, the number of replicated cells remained constant from zygotene to metaphase I, being double the number of replicated cells detected either in premeiosis or in the somatic control (Table 3). Thus, these flow cytometric results clearly confirm that replication occurs during early meiosis in wheat in the absence of the *Ph1* locus, particularly in leptotene and zygotene, and can be monitored and quantified at each stage of meiosis.

### Analysis of the effect of the Ph1 locus on replication during meiosis in wheat

**Figure 3:**
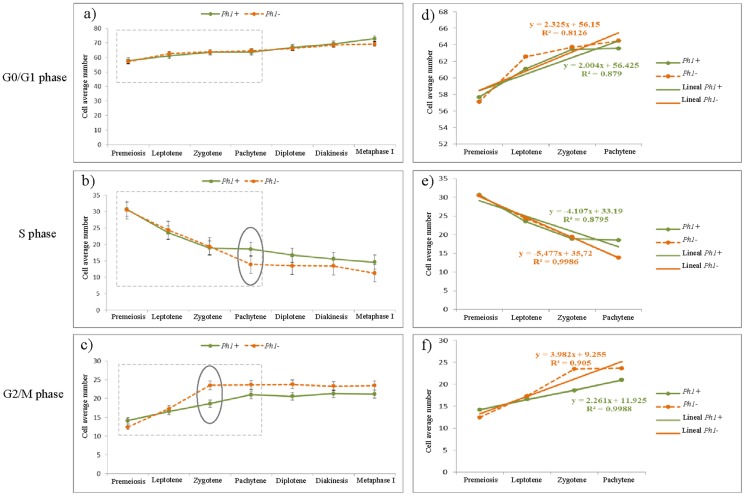
Comparison of the DNA content in wheat anthers in each phase of the cell cycle during the progression of meiosis, in the presence and in the absence of the *Ph1* locus. Each value represents the mean of 9 measurements at each meiotic stage. a) Percentage of cell numbers in each stage of the meiosis in G0/G1 phase of the cell cycle showing no differences in any stage due to the presence/absence of *Ph1* locus. b) Percentage of cell numbers in each stage of the meiosis in S phase. Differences were found in pachytene in the presence and in the absence of the *Ph1* locus. c) Percentage of cell numbers in each stage of the meiosis in G2/M phase. Differences were found in zygotene in the presence and in the absence of the *Ph1* locus. d) Regression line of the values of the percentage of wheat nuclei represented in panel a. No differences were found for the slope of the line either in the presence or in the absence of the *Ph1* locus. e) Regression line of the values of the percentage of wheat nuclei represented in panel b. The slope of the line for replication was higher (in absolute value) in the absence of the *Ph1* locus which means that the rate of replication is higher in its absence. f) Regression line of the values of the percentage of wheat nuclei represented in panel c. The slope of the line was higher in the absence of the *Ph1* locus which implies that new replicated cells appear faster than in the presence of the *Ph1* locus.

The effect of the *Ph1* locus on replication during early meiosis was analysed by flow cytometry. The amount of DNA was measured and compared for each meiotic stage in the presence and in the absence of the *Ph1* locus (Table 4). No differences were found in the number of unreplicated cells (G0/G1 phase) between wheat lines (*Ph1*+, *Ph1*-) during either premeiosis or any stage of prophase I (Figure 3a, Table 4). The only significance differences were found in metaphase I between unreplicated cells of wheat lines in the presence and in the absence of the *Ph1* locus (72.8+0.3 and 69.1+0.6, respectively). These differences may be due to the differences in mature anther size in relation to the presence and absence of the *Ph1* locus, given that anthers in the *ph1* mutant are slightly smaller than anthers in the presence of the *Ph1* locus.

**Table 4:** Comparison of the percentage of nuclei from wheat anthers in each phase of the cell cycle during early meiosis in the presence and in the absence of the *Ph1* locus (*Ph1*+ and *Ph1*-, respectively).

	G0/G1 phase			S phase			G2/M phase		
	Mean ±SE	Mean ±SE	*P*	Mean ±SE	Mean ±SE	*P*	Mean ±SE	Mean ±SE	*P*
	*Ph1+*	*Ph1-*		*Ph1+*	*Ph1-*		*Ph1+*	*Ph1-*	
**Premeiosis**	57.7±2.3a	57.1±0.23a	0.89	30.7±1.2a	30.5±0.9a	0.89	14.1±4.0a	12.4±0.6a	0.12
**Leptotene**	61.1±1.7a	62.6±0.7a	0.44	23.6±1.4a	24.4±1.1a	0.57	16.6±0.8a	17.3±0.2a	0.09
**Zygotene**	63.4±1.4a	63.7±0.1a	0.88	18.9±1.0a	19.4±0.5a	0.06	18.6±0.4a	23.2±0.5b	0.02
**Pachytene**	63.6±0.2a	64.5±0.4a	0.11	18.5±0.8a	13.0±0.9b	0.03	21.0±1.0a	23.6±1.4a	0.28
**Diplotene**	66.8±0.2a	66.1±0.3a	0.11	16.7±0.4a	13.5±0.7a	0.18	20.6±1.3a	23.7±0.3a	0.07
**Diakinesis**	69.2±0.2a	68.3±0.6a	0.36	15.5±1.2a	13.4±0.1a	0.23	21.3±0.6a	23.3±1.3a	0.37
**Metaphase I**	72.8±0.3a	69.1±0.6b	0.02	14.5±0.6a	11.2±0.7a	0.10	21.2±1.5a	23.4±1.5a	0.43

G0/G1 and G2/M values correspond to un-replicated and post-replicated cells, respectively. S phase values correspond to cells under active replication. Values are given as a mean of 9 measurements and standard error of the mean (SE). The same letter indicates no differences among treatments (*Ph1*+ or *Ph1*-) within the same cell cycle phase (G0/G1, G2/M and S) in each stage of meiosis at P<0.05. Differences in replication due to the presence of the *Ph1* locus were only found in pachytene, as replication is still occurring in the presence of the *Ph1* at this stage of meiosis but has finished in its absence.

In contrast, differences in replication during early meiosis in wheat have been revealed by flow cytometric analysis in the presence and in the absence of the *Ph1* locus. Our results showed that at the onset of meiosis replication occurred similarly in both wheat lines with no statistical differences in the amount of DNA either in the presence or in the absence of the *Ph1* locus (Figure 3b, Table 4). Moreover, no significant differences were detected for the S value in early prophase (leptotene and zygotene) between both wheat lines (*Ph1*+ and *Ph1*-) (Figure 3b, Table 4). However, differences in the level of replication were observed in pachytene in wheat in the presence and absence of the *Ph1* locus. Thus, in the presence of the *Ph1* locus replication is still occurring in pachytene (18.5+0.8) meanwhile in the absence of the *Ph1* locus replication had already decreased and reached the basal level (13.0+0.9; Table 4). Therefore, active replication seemed to terminate earlier (zygotene) in wheat in the absence of the *Ph1* locus. After pachytene replication remained similar in both wheat lines (Figure 3b; Table 4).

These data also suggest that there is no significant differences for the G2/M value between wheat lines in the presence and in the absence of the *Ph1* locus in any meiotic stage but in zygotene (Figure 3c, Table 4). The maximum number of replicated cells was reached in zygotene in the absence of the *Ph1* locus meanwhile the number of replicated cells did still increase up to pachytene in the presence of the *Ph1* locus, when the maximum value for the G2/M was reached (Table 4). Therefore, our results indicate that replication is occurring during early meiosis in wheat either in the presence or in the absence of the *Ph1* locus, although differences in the progression of replication have been detected. Our observations suggested that replication timing is affected by the *Ph1* locus as replication finished earlier (zygotene) in the absence of the *Ph1* locus. Moreover, the gradient of the line of the cells number in G0/G1, G2/M and S phases was calculated during early meiosis until pachytene, as no significant differences were found between both lines after this meiotic stage at any time, either in the presence or in the absence of the *Ph1* locus (Figure 3d–f). Results revealed that there are no statistical differences for the slope at the G0/G1 phase due to the presence of the *Ph1* as would be expected. In contrast, differences in the slope of the lines in both the S and the G2/M phases have been observed in the presence and in the absence of the *Ph1* locus. Moreover, the gradient of the line for the S phase was steeper (1.33 times) in the absence of the *Ph1* locus than in its presence, showing that the replication rate is higher in the absence of the *Ph1* locus. As expected, the gradient of the line for the G2/M phase was also higher (1.76 times) in the absence of the *Ph1* than in its presence, indicating that the rate of the increment in the number of replicated cells is higher in the absence of the *Ph1* locus. All these results suggest that replication timing is affected by the presence of the *Ph1* locus, in particular the rate of replication during early meiosis in wheat. The replication rate during meiosis is lower in the presence of the *Ph1* locus. Consequently replication during meiosis in wheat lasts longer in the presence of the *Ph1* locus.

## Discussion

The cell cycle is a much studied process due to its importance in plant growth and development. The significance of replication during the cell cycle is critical to ensure proper chromosome association, recombination and segregation in meiosis, which is directly related to viability of gametes and therefore to fertility. This paper presents a simple and robust method for the determination of the synthesis of DNA during early meiosis by means of flow cytometric measurements in nuclei released from fixed wheat anthers. The synthesis of DNA has been studied using different deoxynucleosides, such as [^3^H] thymidine, 5-bromo-2′-deoxyuridine (BrdU) or 5-ethynyl-2′-deoxyuridine (EdU), which is highly sensitive [46]. Nevertheless, genome size determination, which can be correlated with the synthesis of DNA, must be carried out with a DNA intercalation dye that allows total DNA staining, such as ethidium bromide, propidium iodide, or DAPI (in this work) which provides DNA content histograms with high resolution, uses readily available excitation wavelengths and does not require RNAse treatment of samples. Furthermore, flow cytometry is cheaper than other methods for analysing DNA replication and has already allowed a rapid and accurate analysis of large populations of cells [47]. In fact, flow cytometry has already been applied in plants to determine the nuclear replication stages in seeds from *Lactuca sativa* L., *Solanum melongena* L., and *Lycopersicon esculentatum* Mill., among other species [36], [48]. Fixation of the samples is also often convenient in experiments involving multiple and complex samples. However, fixed nuclear preparations often display wider G1 and G2 peaks in flow cytometric histograms [49]. Nevertheless, although in this work samples were fixed, CV values were below 5% in most of the cases and always lower than the 8%. However, and to the best of our knowledge, it is the first time that this approach is used to quantify replication during meiosis in a crop such as wheat. Replication has been studied in early meiosis in wheat-rye hybrids through the incorporation of EdU [25]. Using flow cytometry we established in this work that DNA synthesis is occurring in early stages of meiosis in common wheat, and quantified the rate of replication during meiosis and the stages of meiosis in which replication occurs. Moreover, using this methodology we have also been able to study the role of the presence of the *Ph1* locus, which controls chromosome pairing [27], on DNA replication during meiosis in wheat. Thus, we observed that chromosome pairing was initiated before the completion of replication, as telomeres started to associate to form a bouquet when replication was still occurring in both wheat lines, either in the presence or in the absence of the *Ph1* locus, similarly to the observations of [25]. But differences in timing of replication during meiosis were found in the presence and in the absence of the wheat *Ph1* locus. Thus replication last longer (until pachytene) during early meiosis in the presence of the *Ph1* locus, or in other words, replication finished earlier (zygotene) when the *Ph1* locus was absent. In fact, our analysis of the slope of the lines at early meiosis indicates that the rate of replication during meiosis is higher in the absence of the *Ph1* locus. Due to the fact that the *Ph1* locus is similar to Cdk2 [29] and Cdk2 affects replication [50], our results confirm one the hypotheses proposed previously [25] by studying wheat-rye hybrids that in the absence of the *Ph1* locus, in that the activation of origins of replication might be increased and consequently the rate of replication.

Residual replication was also detected in wheat anthers at later meiosis stages, after pachytene and zygotene in wheat in the presence and in the absence of the *Ph1* locus, respectively, when chromosomes are associated in pairs. Replication at this stage of meiosis corresponds not only to heterochromatin regions which replicate later in meiosis [25] but also to cell division and therefore residual replication of the somatic surrounding cells.

The structure of chromatin has been shown determinant for the initiation of replication [51]. Moreover, homologous chromosomes usually replicate synchronously although there are some exceptions [52]. In addition, replication of the chromatin has been shown temporary ordered in barley [53]. Our results suggested that sequential replication might be carried out in wheat in the presence of the *Ph1* locus to facilitate correct chromosome associations, and consequently replication takes longer. This could also suggest that only homologous chromosomes are replicating at the same, which could be associated with similar homologous chromosome conformation at early meiosis in wheat, as it has previously described [54], [55]. Finally, our results support previous findings on replication during early meiosis in wheat-rye hybrids in the presence and in the absence of the *Ph1* locus, explained as an increment in the activation of origins and hence the rate of replication of disperse chromatin in wheat-rye hybrids in the absence of the *Ph1* locus [25].

In summary, flow cytometry has been revealed as a suitable tool to detect and quantify DNA replication during early meiosis in wheat. Replication was detected in wheat during premeiosis and early meiosis until the stage of pachytene, when chromosomes are associated in pairs to further recombine and correctly segregate in the gametes. Moreover, flow cytometric results suggested that the *Ph1* locus is affecting the rate of replication during early meiosis in wheat, being lower in the presence of the *Ph1* locus and consequently, replication during early meiosis lasts longer and finishes later than in the absence of the *Ph1* locus. The biological significance of replication at early meiosis and the effect of the *Ph1* locus in replication suggest a solid connection between DNA replication and chromosome associations at the onset of meiosis in a polyploid like wheat. Further studies are needed to build upon these results for unravelling the underlying molecular mechanisms.
